# Bayesian Random Effect Modeling for analyzing spatial clustering of differential time trends of diarrhea incidences

**DOI:** 10.1038/s41598-019-49549-4

**Published:** 2019-09-13

**Authors:** Frank Badu Osei, Alfred Stein

**Affiliations:** Faculty of Geo-Information Science and Earth Observation (ITC), University of Twente, Enschede, Netherlands

**Keywords:** Environmental social sciences, Infectious diseases, Statistics

## Abstract

In 2012, nearly 644,000 people died from diarrhea in sub-Saharan Africa. This is a significant obstacle towards the achievement of the Sustainable Development Goal 3 of ensuring a healthy life and promoting the wellbeing at all ages. To enhance evidence-based site-specific intervention and mitigation strategies, especially in resource-poor countries, we focused on developing differential time trend models for diarrhea. We modeled the logarithm of the unknown risk for each district as a linear function of time with spatially varying effects. We induced correlation between the random intercepts and slopes either by linear functions or bivariate conditional autoregressive (BiCAR) priors. In comparison, models which included correlation between the varying intercepts and slopes outperformed those without. The convolution model with the BiCAR correlation prior was more competitive than the others. The inclusion of correlation between the intercepts and slopes provided an epidemiological value regarding the response of diarrhea infection dynamics to environmental factors in the past and present. We found diarrhea risk to increase by 23% yearly, a rate far exceeding Ghana’s population growth rate of 2.3%. The varying time trends widely varied and clustered, with the majority of districts with at least 80% chance of their rates exceeding the previous years. These findings can be useful for active site-specific evidence-based planning and interventions for diarrhea.

## Introduction

Diarrhea remains a public health menace, and a global obstacle to attain the Sustainable Development Goal 3 (SDG-3) of ensuring healthy lives and promote well-being for all at all ages. Globally, over 1.7 billion episodes of diarrhea are recorded every year with the majority of these occurring in low and middle-income countries^1–5^. The etiological agents, rotavirus, enteropathogenic *E. coli*, enterotoxigenic *E. coli*, calicivirus, and *Shigella*^6,7^, are primarily mediated by environmental, climatic and sociodemographic factors^8^. Children under five years are the most vulnerable^9^. In sub-Saharan Africa, about 644,000 people died from diarrhea in 2012, accounting for 6.7% of deaths. From the 1980s to 2008, diarrhea mortality declined from an estimated 4.5 million to 1.3 million in 2008 with the advent of oral rehydration salts, improved sanitation and access to clean water^1^. Also, the incidences may be declining slightly^4^, yet the statistics are still sobering and unacceptable. Provision of treated water and proper sanitary conditions remains the formidable approach to reducing diarrhea. Budgetary constraints of resource-poor countries make this almost unfeasible. If, however, the spatial patterns of the temporal dynamics are well understood, then limited resources could be channeled to areas experiencing elevated growth trends.

The study of the space-time variation of diarrhea could give critical etiological clues and help to improve resources allocation and planning of interventions. Several studies have addressed issues of spatial and temporal variation of diarrhea with differences in environmental and socioeconomic risk factors, as well as detection of areas with exceptionally high risk^10–14^. Most of these studies, however, either focus on the spatial patterns at a particular point in time or the temporal patterns for an entire geographic area. The reason may be attributable to data challenges and/or unavailable easy to implement statistical methods. Two implicit assumptions are applied under such studies; either the temporal variation of the spatial patterns is assumed to be flat or the spatial variation of the temporal pattern is assumed to be flat. The current advent of information technology has provided opportunities to manage and store geographically and temporally related disease data, at least aggregated over large geographic and temporal windows. Bayesian hierarchical modeling framework offers the advantage to reliably estimate disease parameters through random effects modeling that can be extended to accommodate variation in space, variation in time, and variation in space-time.

In addition to cluster detection methods like the popular space-time scan statistics^15,16^, Bayesian model-based approaches have also found many applications in epidemiology for estimating space-time disease variation due to their flexibility in specifying a variety of spatial, temporal, and space-time interaction structures. Bernardinelli *et al*.^17^ introduced a parametric space-time mapping method to evaluate differential time trends for mapping disease and mortality rates. They assumed a log-linear relationship between the rates and the calendar time within areas and that the time trends vary from area to area. Knorr-Held^18^ extended separable space-time models to include nonparametric space-time interaction effects. The space-time interaction effects are commonly specified either of the four ways: unstructured temporal and unstructured spatial effects (Type I), structured temporal and unstructured spatial effects (Type II), unstructured temporal and structured spatial effects (Type III), or structured temporal and structured spatial effects (Type IV). The common specification for the structured temporal trends for either of these specifications has been the random walk prior. For infectious diseases, areas with similar time trends are likely to form local clusters. To this end, the focus of both Knorr-Held^18^ and Bernardinelli *et al*.^17^, and their various applications have been on providing space-time variation estimates, a divergence from our focus of evaluating the spatial clustering of the differential time trends.

Our aim is to study the spatial clustering of the differential time trends of small area diarrhea occurrences, with the aim of detecting areas of exceptionally high time trends. If there is evidence of spatial clustering of higher than expected temporal trends, specific intervention programs could be developed to target such areas. With our data of few time stamps, a parametric time trend model is decisive. A critical challenge for Bernardinelli *et al*.^17^ was choosing between heterogeneity and clustering, and the way to account for correlation between the varying intercepts and slopes. To address these, we extend their methods to a convolution prior and explore different latent processes to account for correlation between the varying intercepts and slopes. Our data are primarily aggregated counts of yearly diarrhea occurrences for 170 administrative districts for five years. We hypothesize that each district has a diarrhea time trend with spatial random effects, either structured, unstructured or both, that is different from the overall time trend. To achieve this, we used hierarchical Bayesian random effect methods to model the random intercepts and spatially varying time trends jointly. The models we present are extensions of what is presented in^17^. We propose and compare different approaches to account correlations between the spatially varying time trends and intercepts. We develop the models relying on district level diarrhea morbidities in Ghana, where there is limited knowledge of the spatiotemporal trends. Also, despite the declining global trends of diarrhea, morbidities in Ghana continue to increase and remain amongst the top 5 out-patient morbidities. Reported incidences increased from 726,000 cases in 2010 to 1,577,000 cases in 2014. In what follows, we describe the study area and the statistical modeling. Next, we present the results and discuss their implication, and end with some conclusions.

## Methods

### Study area and data

Directly or indirectly, Ghana continues to undertake development projects to increase access to good water and sanitation; some improvements have been achieved over the last few years^19^. However, diarrhea remains amongst the top 5 out-patient morbidities. From 2010 to 2014, incidences increased from 726,000 to 1,577,000 cases. Accordingly, there have been considerable research interests in understanding the etiology, trends, and the characteristics of affected individuals in Ghana^20–27^. Studies of the spatio-temporal trends which remain significant to enhance the optimal usability of scarce resources, however, are scarce. The data used in this study consist of district-level diarrhea morbidities for 170 districts for five years. We obtained the data from the Centre for Health Information and Management (CHIM) of the Ghana Health Services (GHS). We obtained population estimates for the years 2010 to 2014 from the Ghana Statistical Service. The geographical scale of analysis was the 170 administrative districts where data had been recorded.

### Statistical modeling

Bayesian hierarchical modeling easily incorporates correlation processes by including an intermediate layer (*process layer*) between the data likelihood (*data layer*) and the prior distributions (*prior layer*). For the data layer, we consider the spatio-temporal diarrhea counts *y*_*it*_, *i* = 1, …, *m* = 170 districts and *t* = 2010, …, 2014 years as independent random samples from the Poisson distribution, *y*_*it*_|*ς*_*it*_ ~ *Poisson*(*n*_*it*_*ς*_*it*_), where the *ς*_*it*_ is the risk and *n*_*it*_ is the population.

For the process layer, Bernardinelli *et al*.^17^ proposed a monotonically and differentiable log link function to match the risk *ς*_*it*_ and the systematic component, $\log \,{\varsigma }_{it} \sim N({\eta }_{it},{\sigma }_{\varsigma }^{2})$, where

1 $${\eta }_{it}={\beta }_{0}+({\bar{\beta }}_{1}+{\vartheta }_{2i})t+{\vartheta }_{1i}$$

Here, the parameter *β*_0_ denotes the overall intercept (risk) on the log scale, and ${\bar{\beta }}_{1}$ a is fixed effect parameter for the overall time trend in diarrhea growth, while the parameter *ϑ*_2*i*_ is the district-specific spatially structured differential time trends. Inferentially, ${\beta }_{1i}={\bar{\beta }}_{1}+{\vartheta }_{2i}$ specifies the district-specific rate of diarrhea growth. Specifically, *ϑ*_1*i*_ and *ϑ*_2*i*_ are varying intercepts and slopes, respectively. The varying intercepts *ϑ*_1*i*_ account for unobserved ecological factors which might give rise to either spatially structured (**clustering**) or unstructured (**heterogeneity**) extra-Poisson variation.

For a clustering model, the common specification for *ϑ*_1*i*_ or *ϑ*_2*i*_ is as a univariate intrinsic conditional autoregressive (iCAR) process, which depends upon an *m* × *m* spatial proximity matrix *w*_*ij*_ with unknown variance ${\sigma }_{\vartheta }^{2}$. The iCAR specification implies that the distribution of *ϑ*_***ki***_ = {*ϑ*_1*i*_, *ϑ*_2*i*_} conditional on the set *ϑ*_*k*,−*i*_ = *ϑ*_*k*,*j*≠*i*_ = {*ϑ*_*k*1_, …, *ϑ*_*k*,*i*−1_,*ϑ*_*k*,*i*+1_, …, *ϑ*_*km*_} are weighted averages of function evaluations of *J* neighboring districts; thus ${\vartheta }_{ki}|{\vartheta }_{k,-i} \sim N({\bar{\vartheta }}_{ki},{\sigma }_{k\vartheta }^{2}/{\sum }_{j\ne i}{w}_{ij})$, *k* = 1, 2. The mean ${\bar{\vartheta }}_{ki}={\sum }_{j}{w}_{ij}{\vartheta }_{kj}/{\sum }_{j\ne i}{w}_{ij}$, ∀*j* ∈ *w*_*ij*_, where *w*_*ij*_, *j* = 1, …, *J* denotes the set of *J* neighbors of districts *i*. The weights *w*_*ij*_ are fixed constants that measure the proximity of districts *i*and *j*. Let the set of boundary points on district *i* be denoted by (*i*). Then we define *w*_*ij*_ as a binary connectivity weight matrix such that *w*_*ij*_ = 1 if $(i)\cap (j)\ne \varnothing$, and *w*_*ij*_ = 0 otherwise. For brevity, we write the iCAR prior as ${\vartheta }_{ki} \sim iCAR({\bar{\vartheta }}_{ki},{\sigma }_{{\vartheta }_{k}}^{2})$. Since iCAR is translational invariant, the constraints ${\sum }_{i}{\vartheta }_{ki}=0$ is required for identifiability of the mean.

Choosing between heterogeneity and clustering of the time trends and/or the intercepts is critical. The choice depends upon the prior belief about the scale of spatial dependency. A spatial scale of dependency larger (smaller) than the size of spatial units leads to clustering (heterogeneity). For infectious diseases, both the smaller and larger scale of spatial dependency is plausible. To avoid choosing between these two, we propose to use a convolution model which includes both clustering and heterogeneous random intercepts and slopes:

2 $${\eta }_{it}={\beta }_{0}+({\bar{\beta }}_{1}+{\vartheta }_{2i}+{\upsilon }_{2i})t+{\vartheta }_{1i}+{\upsilon }_{1i}$$

where *ϑ*_*ki*_ = {*ϑ*_1*i*_, *ϑ*_2*i*_} are modeled as iCAR processes ${\vartheta }_{ki} \sim iCAR({\bar{\vartheta }}_{ki},{\sigma }_{{\vartheta }_{k}}^{2})$ with unknown prior variance ${\sigma }_{{\vartheta }_{k}}^{2}$ for the clustering components and *υ*_*ki*_ = {*υ*_1*i*_, *υ*_2*i*_} are assigned Gaussian processes ${\upsilon }_{ki} \sim N(0,{\sigma }_{{\upsilon }_{k}}^{2})$ with unknown prior variance ${\sigma }_{{\upsilon }_{k}}^{2}$ for the heterogeneity components. Under this prior structure, the ${\upsilon }_{ki} \sim N(0,{\sigma }_{{\upsilon }_{k}}^{2})$ do not depend upon the structure of spatial units and are said to be exchangeable. Specifying ${\vartheta }_{2i} \sim N(0,{\sigma }_{{\vartheta }_{2}}^{2})$ implies random exchangeability between the differential time growths, whereas ${\vartheta }_{2i} \sim iCAR({\bar{\vartheta }}_{2i},{\sigma }_{{\vartheta }_{2}}^{2})$ implies Markovian interactions between the differential time growths. The expression ${\vartheta }_{2i} \sim iCAR({\bar{\vartheta }}_{2i},{\sigma }_{{\vartheta }_{2}}^{2})$ then becomes a model for the district-specific time trends.

### Correlation between random intercepts and slopes

As it stands now, the latent variables ${\vartheta }_{ki} \sim iCAR({\bar{\vartheta }}_{ki},{\sigma }_{{\vartheta }_{k}}^{2})$ and ${\upsilon }_{ki} \sim N(0,{\sigma }_{{\upsilon }_{k}}^{2})$ are independent. For epidemiological purposes, allowing for correlation between *ϑ*_1*i*_ and *ϑ*_2*i*_, and that of *υ*_1*i*_ and *υ*_2*i*_ could answer critical etiological questions such as how the population has reacted towards environmental factors introduced at the reference time of the study. Avoiding the correlation between the random intercepts and slopes of the time trends could cause the areas-specific time tends to be pulled towards the overall mean trend^17^. For models such as (1), and when heterogeneity modeling is the concern, Bernardinelli *et al*.^17^ proposed *ϑ*_1*i*_ as drawn by a univariate normal ${\upsilon }_{1i} \sim N(0,\,{\sigma }_{{\upsilon }_{1}}^{2})$, while *υ*_2*i*_ is also drawn from a univariate normal conditional on *ϑ*_1*i*_, ${\upsilon }_{2i}|{\upsilon }_{1i} \sim N({\gamma }_{\upsilon }{\upsilon }_{1i},\,{\sigma }_{{\upsilon }_{2}}^{2})$. Here, *γ*_*υ*_ is the correlation parameter between the unstructured intercepts *υ*_1*i*_ and slopes *υ*_2*i*_. With this premise, other variants can be deduced. For instance, when clustering is of concern for both slopes and intercepts, then the latent variable *ϑ*_1*i*_ is modeled as drawn by the conditional autoregressive process ${\vartheta }_{1i} \sim iCAR({\bar{\vartheta }}_{1i},{\sigma }_{{\vartheta }_{1}}^{2})$, and *ϑ*_2*i*_ as drawn from a univariate normal conditional on *ϑ*_1*i*_, ${\vartheta }_{2i}|{\vartheta }_{1i} \sim N({\gamma }_{\vartheta }{\vartheta }_{1i},\,{\sigma }_{{\vartheta }_{2}}^{2})$. Besides, when both clustering and heterogeneity are of concern, correlation can be induced by expressing ${\vartheta }_{1i} \sim iCAR({\bar{\vartheta }}_{1i},\,{\sigma }_{{\vartheta }_{1}}^{2})$, ${\upsilon }_{1i} \sim N(0,\,{\sigma }_{{\upsilon }_{1}}^{2})$, and $({\vartheta }_{2i}+{\upsilon }_{2i})|{\vartheta }_{1i},{\upsilon }_{1i} \sim N({\gamma }_{\upsilon \vartheta }\{{\vartheta }_{1i}+{\upsilon }_{1i}\},\,{\sigma }_{{\vartheta }_{2}+{\upsilon }_{2}}^{2})$, where ${\sigma }_{{\vartheta }_{2}+{\upsilon }_{2}}^{2}$ is the total variance of *ϑ*_1*i*_ + *υ*_1*i*_. An additional possibility is to account for correlations separately between the structured and unstructured intercepts and slopes by modeling the latent variables ${\vartheta }_{1i} \sim iCAR({\bar{\vartheta }}_{1i},\,{\sigma }_{{\vartheta }_{1}}^{2})$, ${\upsilon }_{1i} \sim N(0,\,{\sigma }_{{\upsilon }_{1}}^{2})$, ${\vartheta }_{2i}|{\vartheta }_{1i} \sim N({\gamma }_{\vartheta }{\vartheta }_{1i},{\sigma }_{{\vartheta }_{2}}^{2})$, ${\upsilon }_{2i}|{\upsilon }_{1i} \sim N({\gamma }_{\upsilon }{\upsilon }_{1i},\,{\sigma }_{{\upsilon }_{2}}^{2})$. For the correlation parameters *γ*_*υ*_,*γ*_*ϑ*_, and *γ*_*υϑ*_, samples may be drawn from the univariate normal distributions ${\gamma }_{\upsilon } \sim N(0,\,{\sigma }_{{\gamma }_{\upsilon }}^{2})$, ${\gamma }_{\vartheta } \sim N(0,\,{\sigma }_{{\gamma }_{\vartheta }}^{2})$, and ${\gamma }_{\upsilon \vartheta } \sim N(0,\,{\sigma }_{{\gamma }_{\upsilon \vartheta }}^{2})$, respectively.

We propose to specify the bivariate iCAR (BiCAR) for the clustering components *ϑ*_*ki*_ = {*ϑ*_1*i*_, *ϑ*_2*i*_}. The univariate iCAR extends naturally to the BiCAR specification by replacing the univariate normal condi-tional distribution with a bivariate conditional distribution ${\vartheta }_{ki}|({\vartheta }_{1,-i},{\vartheta }_{2,-i}) \sim N({\bar{\vartheta }}_{ki},{\Sigma }_{\vartheta }/{\sum }_{j\ne i}{w}_{ij})$ where ${\bar{\vartheta }}_{ki}={({\sum }_{j}{w}_{ij}{\vartheta }_{1j}/{\sum }_{j\ne i}{w}_{ij},{\sum }_{j}{w}_{ij}{\vartheta }_{2j}/{\sum }_{j\ne i}{w}_{ij})}^{{\boldsymbol{T}}}$ is the mean vector and Σ_*ϑ*_ is a 2 × 2 covariance matrix. The covariance Σ_*ϑ*_ has diagonal elements ${\Sigma }_{\vartheta }[1,1]={\sigma }_{{\vartheta }_{1}}^{2}$ and ${\Sigma }_{\vartheta }[2,2]={\sigma }_{{\vartheta }_{2}}^{2}$ representing the conditional variances for the structured *ϑ*_2*i*_ slopes and intercept *ϑ*_1*i*_, respectively. The off-diagonal elements ${\Sigma }_{\vartheta }[1,2]={\Sigma }_{\vartheta }[2,1]={\gamma }_{\vartheta }{\sigma }_{{\vartheta }_{1}}^{2}{\sigma }_{{\vartheta }_{2}}^{2}$ captures the within-area correlation through the correlation parameter *γ*_*ϑ*_. For brevity, we write ${\vartheta }_{ki} \sim BiCAR({\bar{\vartheta }}_{ki},{\Sigma }_{\vartheta })$. For the heterogeneity components *υ*_*ki*_ = {*υ*_1*i*_, *υ*_2*i*_}, we propose to use a zero-mean bivariate normal distribution *υ*_*ki*_ ~ *N*_2_(0, Σ_*υ*_) where Σ_*υ*_ is a 2 × 2 covariance matrix. The specification for Σ_*υ*_ follows as Σ_*ϑ*_ described above where ${\Sigma }_{\upsilon }[1,1]={\sigma }_{{\upsilon }_{1}}^{2}$, ${\Sigma }_{\upsilon }[2,2]={\sigma }_{{\upsilon }_{2}}^{2}$, and ${\Sigma }_{\upsilon }[1,2]={\Sigma }_{\upsilon }[2,1]={\gamma }_{\upsilon }{\sigma }_{{\upsilon }_{1}}^{2}{\sigma }_{{\upsilon }_{2}}^{2}$. Here, *γ*_*υ*_ is the within-area correlation between the unstructured intercepts and slopes.

For the third layer, the prior layer, we assign prior distributions to all variance parameters and fixed effects. A non-informative flat distribution for the intercept, *p*(*β*_0_) ∝ 1, is appropriate to ensure that the data drive inference. Non-informative priors result in posterior inference similar to maximum likelihood inference^28^. For the fixed effects, vague normal priors are specified ${\bar{\beta }}_{1}$, ${\bar{\beta }}_{1} \sim N(0,{10}^{5})$. This is the equivalent to a non-informative Gaussian prior. To the variance parameters ${\sigma }^{2}=\{{\sigma }_{{\vartheta }_{k}}^{2},{\sigma }_{{\upsilon }_{k}}^{2},{\sigma }_{\varsigma }^{2}\}$, we assigned proper vague gamma priors *σ*^−2^ ~ *G*(0.5, 0.05) to ensure conjugacy and computational convenience^29^. We assigned a Wishart prior to the two precision matrices ${\Sigma }_{\vartheta }^{-1}$ and ${\Sigma }_{\upsilon }^{-1}$, as it is a conjugate prior for the inverse of the covariance parameters of a multivariate normal distribution^30,31^. More precisely, we assigned ${\Sigma }_{\vartheta }^{-1} \sim {\rm{Wishart}}(\Omega ,df)$, ${\Sigma }_{\upsilon }^{-1} \sim {\rm{Wishart}}(\Omega ,df)$ with scale matrix Ω and degrees of freedom *df* = 2 for a weakly informative distribution. We set Ω as a scaled identity matrix with diagonal entries Ω[1, 1] = Ω[2, 2] = 1 and off-diagonal entries Ω[1, 2] = Ω[2, 1] = 0, a specification Moraga and Lawson^32^ utilized to run simulation studies of multivariate iCAR modeling.

### Model fitting and estimation

We fitted eight different models with different combinations and structures of the process layer (Table 1). Models 1 to 3 include no correlation between the intercepts and slopes while Models 4 to 8 include different forms of interaction between the intercepts and slopes. Model 1 is a spatially varying unstructured time coefficients model with unstructured spatial effects. Model 2 includes an iCAR structured time-varying coefficients and structured iCAR intercepts. Model 3 is a convolution model which includes spatially varying unstructured and structured time effects, and unstructured and structured intercepts. Model 4 and Model 5 extend Model 1 and Model 2, respectively, to include correlation between the intercepts and slopes. In Model 6, we induce correlation by expressing the sum of structured and unstructured time coefficients as a linear function of the sum of structured and unstructured intercepts. In Model 7, we induce separate correlations between the structured intercepts and slopes and the unstructured intercepts and slopes. Finally, Model 8, uses a BiCAR specification to account for correlation between the structured incepted and slopes and a multivariate normal distribution to account for correlation between the unstructured intercepts and slopes. In Table 1, we present details of the models and structures of the latent parameters and their prior distributions.

**Table 1:** Latent structures of the different models.

Model	Linea predictor *η*_*it*_	Structured	Unstructured	correlation
1	${\beta }_{0}+({\bar{\beta }}_{1}+{\upsilon }_{2i})t+{\upsilon }_{1i}$		${\upsilon }_{ki} \sim N(0,{\sigma }_{{\upsilon }_{k}}^{2})$ $k=1,2$	
2	${\beta }_{0}+({\bar{\beta }}_{1}+{\vartheta }_{2i})t+{\vartheta }_{1i}$	${\vartheta }_{ki} \sim iCAR({\bar{\vartheta }}_{ki},{\sigma }_{{\vartheta }_{k}}^{2})$ *k* = 1, 2		
3	${\beta }_{0}+({\bar{\beta }}_{1}+{\vartheta }_{2i}+{\upsilon }_{2i})t+{\vartheta }_{1i}+{\upsilon }_{1i}$	${\vartheta }_{ki} \sim iCAR({\bar{\vartheta }}_{ki},{\sigma }_{{\vartheta }_{k}}^{2})$ *k* = 1, 2	${\upsilon }_{ki} \sim N(0,{\sigma }_{{\upsilon }_{k}}^{2})$ *k* = 1, 2	
4	${\beta }_{0}+({\bar{\beta }}_{1}+{\upsilon }_{2i})t+{\upsilon }_{1i}$		${\upsilon }_{1i} \sim N(0,\,{\sigma }_{{\vartheta }_{1}}^{2})$ ${\upsilon }_{2i}|{\upsilon }_{1i} \sim N(\gamma {\upsilon }_{1i},\,{\sigma }_{{\upsilon }_{2}}^{2})$	${\gamma }_{\upsilon } \sim N(0,{\sigma }_{{\gamma }_{\upsilon }}^{2})$
5	${\beta }_{0}+({\bar{\beta }}_{1}+{\vartheta }_{2i})t+{\vartheta }_{1i}$	${\vartheta }_{1i} \sim iCAR({\bar{\vartheta }}_{1i},\,{\sigma }_{{\vartheta }_{1}}^{2})$ ${\vartheta }_{2i}|{\vartheta }_{1i} \sim N(\gamma {\vartheta }_{1i},\,{\sigma }_{{\vartheta }_{2}}^{2})$		${\gamma }_{\vartheta } \sim N(0,{\sigma }_{{\gamma }_{\vartheta }}^{2})$
6	${\beta }_{0}+({\bar{\beta }}_{1}+{\vartheta }_{2i}+{\upsilon }_{2i})t+{\vartheta }_{1i}+{\upsilon }_{1i}$	${\vartheta }_{1i} \sim iCAR({\bar{\vartheta }}_{1i},\,{\sigma }_{{\vartheta }_{1}}^{2})$ *ϑ*_2*i*_ + *υ*_2*i*_|*ϑ*_1*i*_,*υ*_1*i*_~ $N({\gamma }_{\vartheta \upsilon }({\vartheta }_{1i}+{\upsilon }_{1i}),\,{\sigma }_{{\vartheta }_{2}+{\upsilon }_{2}}^{2})$	${\upsilon }_{1i} \sim N(0,\,{\sigma }_{{\upsilon }_{1}}^{2})$	${\gamma }_{\vartheta \upsilon } \sim N(0,\,{\sigma }_{{\gamma }_{\vartheta \upsilon }}^{2})$
7	${\beta }_{0}+({\bar{\beta }}_{1}+{\vartheta }_{2i}+{\upsilon }_{2i})t+{\vartheta }_{1i}+{\upsilon }_{1i}$	${\vartheta }_{1i} \sim iCAR({\bar{\vartheta }}_{1i},\,{\sigma }_{{\vartheta }_{1}}^{2})$ ${\vartheta }_{2i}|{\vartheta }_{1i} \sim N({\gamma }_{\vartheta }{\vartheta }_{1i},{\sigma }_{{\vartheta }_{2}}^{2})$	${\upsilon }_{1i} \sim N(0,\,{\sigma }_{{\upsilon }_{1}}^{2})$ ${\upsilon }_{2i}|{\upsilon }_{1i} \sim N({\gamma }_{\upsilon }{\upsilon }_{1i},\,{\sigma }_{{\upsilon }_{2}}^{2})$	${\gamma }_{\vartheta } \sim N(0,{\sigma }_{{\gamma }_{\vartheta }}^{2})$ ${\gamma }_{\upsilon } \sim N(0,{\sigma }_{\upsilon }^{2})$
8	${\beta }_{0}+({\bar{\beta }}_{1}+{\vartheta }_{2i}+{\upsilon }_{2i})t+{\vartheta }_{1i}+{\upsilon }_{1i}$	${\vartheta }_{ki} \sim BiCAR({\bar{\vartheta }}_{ki},{\Sigma }_{\vartheta })$	*υ*_*ki*_ ~ *N*_2_(**0**, Σ*υ*)	*γ*_*ϑ*_, *γ*_*υ*_, *γ*_*ϑυ*_

We estimated the parameters of the models within the Bayesian hierarchical framework. For each model, let the vector ψ_1_ be a full Gaussian latent field that represents the process layer, and ψ_2_ represent the prior layer, then the joint density *p*(ψ_1_, ψ_2_) = *p*(ψ_1_|ψ_2_)*p*(ψ_2_). Following the Bayesian paradigm, we factorize the posterior density *p*(ψ_1_|ψ_2_|y) as proportional to the product of the data layer *p*(y|ψ_1_, ψ_2_) and the joint density *p*(ψ_1_, ψ_2_), $p({{\rm{\psi }}}_{1}{|{\rm{\psi }}}_{2}|y)\propto p({\rm{y}}|{{\rm{\psi }}}_{1},{{\rm{\psi }}}_{2})\cdot p({{\rm{\psi }}}_{1}|{{\rm{\psi }}}_{2})\cdot p({{\rm{\psi }}}_{2})$. We used Markov Chains Monte Carlo (MCMC) simulations to draw samples from the full conditional density of the posterior *p*(ψ_1_|ψ_2_|y). Estimation was implemented in the WINBUGS 1.4.3 software package^33^. We used 200,000 MCMC iterations and 100,000 burn-in samples, storing only every 20th sampled parameter of the Markov chains. We implemented the model using three independent chains with dispersed initial values. Convergence was assessed graphically by the autocorrelation plots of the traces. We fitted all models using the R2WinBUGS package^33^ together with the R software^34^. Point estimates of variables of interest and their associated uncertainties were obtained via the marginal posterior distributions.

### Model evaluation and comparison

We evaluated the adequacy of the model fits using two different cross-validating predictive checks. First, we used the posterior predictive checks and Bayesian *p*-values, defined as ${\rm{\Pr }}(\,{y}_{it}\ge {y}_{it}^{pred})$, where the predicted datasets ${y}_{it}^{\,pred}$ are generated from the predictive distribution of the models. Bayesian *p*-values close to 0.5 suggests that the generated data are compatible with the model, whereas values close to 0 and 1 are considered extreme and hence suggest a poor fit. Since the distribution of the Bayesian *p*-values is not symmetrical, values <0.1 and >0.9 were considered extreme values and, hence, an indication of poor fit^35^. Additionally, we used the Chi-square goodness-of-fit statistic based on the discrepancy function, ${\chi }_{obs}^{2}={\sum }_{it}[{({y}_{it}-{n}_{it}{\varsigma }_{it})}^{2}/{n}_{it}{\varsigma }_{it}]$^36^. Similarly, ${\chi }_{pred}^{2}$ is calculated for the predicted datasets ${y}_{it}^{\,pred}$. The two quantities are compared using Bayesian *p*-values, here, defined as ${\rm{\Pr }}({\chi }_{obs}^{2}\ge {\chi }_{pred}^{2})$. Likewise, Bayesian *p*-values close to 0.5 suggests that the generated data are compatible with the model.

We compared the models using the deviance information criterion (DIC). The $\,DIC=\bar{D}+{p}_{D}$ is the sum of the model fit $\bar{D}$ and model complexity *p*_*D*_^37^. Negative twice the log-likelihood of the deviance informs the model fit, while the effective number of parameters informs the model complexity. It is a generalization of the Akaike’s information criterion (AIC). Like the AIC, the smaller the DIC value, the better the predictive performance of the model.

### Posterior estimates

We estimated the posterior means of the parameters from the posterior samples. For the parameters ${\hat{\gamma }}_{\vartheta }$, ${\hat{\gamma }}_{v}$, and ${\hat{\gamma }}_{\vartheta v}$ of Model 8, empirical analogs were used. From the posterior estimates of the covariance matrices ${\hat{\Sigma }}_{\vartheta }$ and ${\hat{\Sigma }}_{\upsilon }$, we estimated the within-area correlations between the structured slopes and intercepts. Let ${\hat{\sigma }}_{{\vartheta }_{1}}^{2}={\hat{\Sigma }}_{\vartheta }[1,1]$, ${\hat{\sigma }}_{{\vartheta }_{2}}^{2}={\hat{\Sigma }}_{\vartheta }[2,2]$, ${\hat{\sigma }}_{{\upsilon }_{1}}^{2}={\hat{\Sigma }}_{\upsilon }[1,1]$, ${\hat{\sigma }}_{{\upsilon }_{2}}^{2}={\hat{\Sigma }}_{\upsilon }[2,2]$ be the empirical variances of the structured and unstructured time effects and slopes, respectively. Then ${\hat{\gamma }}_{\vartheta }={\hat{\Sigma }}_{\vartheta }[1,2]/{\hat{\sigma }}_{{\vartheta }_{1}}{\hat{\sigma }}_{{\vartheta }_{2}}$ is the empirical estimate of the correlations between the structured slopes and intercepts and ${\hat{\gamma }}_{\upsilon }={\hat{\Sigma }}_{\upsilon }[1,2]/{\hat{\sigma }}_{{\upsilon }_{1}}{\hat{\sigma }}_{{\upsilon }_{2}}$ for that of the unstructured slopes and intercepts. The total within-area correlation between the slopes and intercepts equals ${\hat{\gamma }}_{\vartheta v}=({\hat{\Sigma }}_{\vartheta }[1,2]+{\hat{\Sigma }}_{\upsilon }[1,2])/\sqrt{{\hat{\sigma }}_{{\vartheta }_{1}}^{2}+{\hat{\sigma }}_{{\upsilon }_{1}}^{2}}\sqrt{{\hat{\sigma }}_{{\vartheta }_{2}}^{2}+{\hat{\sigma }}_{{\upsilon }_{2}}^{2}}$.

Next, we estimated the relative contribution of the structured and unstructured slopes and intercepts as fractions of the marginal variability of ${\sigma }_{{\vartheta }_{k}}^{2}$ over the total marginal variability ${\sigma }_{{\vartheta }_{k}}^{2}+{\sigma }_{{\upsilon }_{k}}^{2}$. Since the parameter ${\sigma }_{{\vartheta }_{k}}^{2}$ is not directly available, we used its empirical analog ${\hat{\sigma }}_{{\vartheta }_{k}}^{2}=\sum _{i}({\vartheta }_{ki}-{\bar{\vartheta }}_{k})/(n-1)$. Thus, $fra{c}_{{\vartheta }_{k}}={\hat{\sigma }}_{{\vartheta }_{k}}^{2}/{\sigma }_{{\vartheta }_{k}}^{2}+{\sigma }_{{\upsilon }_{k}}^{2})$ is the relative contribution of spatially structured slope and intercepts.

### Spatial clustering of time trends

Our critical interest was on the spatial clustering of the differential time trends. To evaluate this, we dwell on the uncertainties or posterior probabilities associated with the posterior means of the district-specific time trends *β*_1*i*_. We used the spatially smoothed exceedance probabilities of the posterior measures to detect and map clustering of these parameters instead of their raw estimates. First, from the posterior samples ${\{{\beta }_{1i}^{g}\}}_{g=1,\ldots ,G}$ of say *β*_1*i*_, we estimated the exceedance probabilities ${\rm{\Pr }}({\beta }_{1i} > {\beta }_{1}^{Th})$ as the probability that *β*_1*i*_ exceeds some threshold level say ${\beta }_{1}^{Th}$. We estimated $\,\Pr ({\beta }_{i1} > {\beta }_{1}^{Th})$ as how frequently the posterior samples ${\{{\beta }_{1i}^{g}\}}_{g=1,\ldots ,G}$ exceed the threshold ${\beta }_{1}^{Th}$ during the MCMC iterations. We then define $\,\Pr ({\beta }_{1i} > {\beta }_{1}^{Th})=\mathop{\sum }\limits_{g=1}^{G}I({\beta }_{1i}^{g} > {\beta }_{1}^{Th})/G$, where *I*() is the indicator function. Next, we estimated the spatially smoothed posterior probabilities as $\overline{{\rm{\Pr }}}({\beta }_{1i} > {\beta }_{1}^{Th})=\mathop{\sum }\limits_{j=0}^{J}{\rm{\Pr }}({\beta }_{1,ij} > {\beta }_{1}^{Th})/(J+1)$, ∀*j* ∈ *w*_*ij*_, where ${\rm{\Pr }}({\beta }_{1,i0} > {\beta }_{1}^{Th})={\rm{\Pr }}({\beta }_{1i} > {\beta }_{1}^{Th})$. We set the threshold at ${\beta }_{1}^{Th}=\bar{\beta }$. We checked the sensitivity of the exceedances by estimating the probabilities for different incremental multiplicative effects $\exp ({\beta }_{1}^{Th})=\{\exp (1.0,\,1.10,\,1.20,\,1.25,\,2.0\}$. Also for the random effects, *ϑ*_1*i*_, *υ*_1*i*_, *ϑ*_2*i*_, *υ*_2*i*_, we similarly estimated the spatially smoothed exceedance probabilities $\Pr (\exp ({\vartheta }_{1i}) > 1)$, $\Pr (\exp ({\vartheta }_{2i}) > 1)$, $\Pr (\exp ({\upsilon }_{1i}) > 1)$, and $\Pr (\exp ({\upsilon }_{2i}) > 1)$.

## Results and Analyses

The adequacy of the models was evaluated using Bayesian predicted checks and the chi-square goodness-of-fit test. Table 2 provides estimates of the model fit parameters. An adequate fit is suggested for all the models as the ${p}_{Bayes}^{\chi }$ for the chi-square goodness-of-fit test lies within the interval [0.1, 0.9]^35^. On the mean of the Bayesian *p*-values of the predictive checks, all models obtained ${\bar{p}}_{Bayes}^{pred}=0.5$, also supporting adequate fit. Additional confirmation of adequate model fit is also based on the fact that just less than 3% of the observations had extreme Bayesian *p*-value, $\% {p}_{Bayes}^{ext\_pred} < 3 \%$.

**Table 2:** Parameters of Models fit and comparison.

Parameters	Model 1	Model 2	Model 3	Model 4	Model 5	Model 6	Model 7	Model 8
${\chi }_{obs}^{2}$	901.96	905.77	904.13	899.45	885.83	898.62	897.31	896.03
${\chi }_{rep}^{2}$	832.96	831.26	834.25	832.39	833.29	832.70	831.87	832.03
${p}_{Bayes}^{\chi }$	0.87	0.89	0.89	0.88	0.82	0.86	0.88	0.86
${\bar{p}}_{Bayes}^{pred}$	0.5	0.5	0.5	0.5	0.5	0.5	0.5	0.5
$\% {p}_{Bayes}^{ext\_pred}$	1.76	1.41	1.65	2.11	0.71	1.18	1.76	0.94
*Gamma priors* (shape, rate)	*DIC*							
(0.5, 0.0005)	10668.2	10658.2	10653.1	10646.5	10647.0	10645.9	10640.8	10626.8
(0.5, 0.005)	10669.4	10659.4	10652.4	10644.7	10644.5	10644.9	10641.6	10628.6
(0.5, 0.05)	**10667.2**	**10656.2**	**10651.0**	**10642.8**	**10644.4**	**10644.0**	**10640.1**	**10627.5**
(0.5, 0.5)	10667.5	10657.5	10651.7	10645.8	10648.5	10645.6	10641.7	10627.8

We studied the sensitivity of our results to various gamma priors for the variance parameters. We specifically varied the rate parameter in the gamma prior while maintaining the shape parameter. These results are shown in Table 2. Under the different gamma priors, the DIC values for each model were only marginally different, suggesting that the results are not overly sensitive to the choice of gamma priors. Analyzing the DIC values from Table 2 for model comparison, the significance of including correlation between the random intercepts and slopes can be observed in Models 4 to 8. The DIC values of these models decrease as compared to Models 1 to 3. This indicates that the possible correlation between the random slopes and intercepts should not be overlooked. Models 4 to 8 each have their weakness and strengths in terms of accounting for the correlations. Unlike Models 4 to 6 which are able to either account for correlation between unstructured intercepts and slopes, or structured intercepts and slopes or both, Models 7 and 8 evaluate joint correlations between the unstructured intercepts and slopes and between the structured intercepts and slopes. Comparing the DIC values, Model 8 provides an improvement over all the other models indicating that multivariate structures on the intercepts *ϑ*_*ki*_ and slopes *υ*_*ki*_ best support our data. The advantage of specifying *ϑ*_*ki*_ and *υ*_*ki*_ as multivariate structures is noticeable in capturing the separate correlations; thus ${\hat{\gamma }}_{{\vartheta }_{1,2}}$ between *ϑ*_1*i*_ and *ϑ*_2*i*_, ${\hat{\gamma }}_{{\upsilon }_{1,2}}$ between *υ*_1*i*_ and *υ*_2*i*_, and the total correlation ${\hat{\gamma }}_{\vartheta v}$. Yet, Model 7 is also appealing regarding its simple structure. In our implementation, the data showed no significant correlation between the structured slopes and intercepts for Models 7 and 8.

Table 3 reports the posterior estimates of the parameters of all models. We observe consistent estimates for most of the parameters, though differences are observed for some parameters. The overall incidence rate is $\exp ({\beta }_{0})\approx 4.5$ per 100 people for all the models. For the time trends, all models showed $\exp ({\bar{\beta }}_{1})\approx 1.23$. This corresponds to 23% yearly average increases in diarrhea risk in Ghana. Unlike the variances of the structured and unstructured intercepts ${\sigma }_{{\vartheta }_{1}}^{2}$, ${\sigma }_{{\upsilon }_{1}}^{2}$ and slopes ${\sigma }_{{\vartheta }_{2}}^{2}$, ${\sigma }_{{\upsilon }_{2}}^{2}$, the differences in the posterior estimates of the between-districts variances of the risks ${\sigma }_{\varsigma }^{2}$ are marginal. This indicates that the district-specific risk estimates are robust to the choice of model and latent structures for smoothing. Thus, little overall smoothing is performed. The variances, on the other hand, are sensitive to the choice of latent smoothing structures imposed on the varying intercepts and lopes. We observe, however, similar variances amongst the varying intercepts or slopes for some models where the correlation parameter is the only difference. For instance, Models 1 and 4 have marginal differences in their variances for the intercepts but not for the slopes. The same applies to Models 2 and 5, suggesting that the intercepts parameters are rather robust when the correlation between intercepts and slopes are accounted for in this manner. For the models with either structured or unstructured random effects (intercepts and slopes or both), the unstructured components always dominate ($fra{c}_{{\vartheta }_{2}} < 50 \%$ and $fra{c}_{{\vartheta }_{2}} < 50 \%$). This suggests the dominance of heterogeneity over clustering. Model 6 accounts for the correlation between the convolution intercepts and slopes. We observe the same correlation parameter estimate as Models 4 and 5, with a similar variance for the structured intercepts of its variant model (Model 3), except for the structured intercepts component, which is largely reduced. This is an indication of the robustness of the convolution method of accounting for correlation between the random slopes and intercepts. Results of Models 7 and 8 indicate similar estimates for most variance components since they both account for separate correlations between the unstructured and structured intercepts and slopes.

**Table 3:** Posterior estimates of model parameters.

Parameters	Without correlation			With correlation				
	Model 1	Model 2	Model 3	Model 4	Model 5	Model 6	Model 7	Model 8
$exp({\beta }_{0})$	0.0449	0.0449	0.0452	0.0446	0.0449	0.0452	0.0450	0.0450
$exp({\bar{\beta }}_{1})$	1.2318	1.2332	1.2322	1.2339	1.2338	1.2299	1.2333	1.2353
${\sigma }_{\varsigma }^{2}$	0.1673 (0.0111)	0.1744 (0.0114)	0.1679 (0.0111)	0.1689 (0.0107)	0.1711 (0.0115)	0.1690 (0.0111)	0.1693 (0.0110)	0.1667 (0.0107)
${\sigma }_{{\vartheta }_{1}}^{2}$	*	1.4375 (0.1805)	0.1166 (0.0846)	*	1.4961 (0.1856)	0.0699 (0.0529)	0.0764 (0.0586)	0.2117 (0.0854)
${\sigma }_{{\vartheta }_{2}}^{2}$	*	0.1802 (0.0310)	0.0227 (0.0209)	*	0.0386 (0.0386)	*	0.0104 (0.0122)	0.0717 (0.0186)
${\sigma }_{{\upsilon }_{1}}^{2}$	0.327 (0.038)	*	0.2633 (0.0464)	0.3253 (0.039)	*	0.2799 (0.0404)	0.2757 (0.0390)	0.2528 (0.0395)
${\sigma }_{{\upsilon }_{2}}^{2}$	0.049 (0.007)	*	0.0383 (0.0089)	0.0383 (0.0062)	*	*	0.0262 (0.0129)	0.0495 (0.0079)
${\sigma }_{{\vartheta }_{2}+{\upsilon }_{2}}^{2}$	*	*	*	*	*	0.0381 (0.0065)	*	*
${\hat{\gamma }}_{\vartheta }$	*	*	*	*	−0.1827 (0.0346)	*	−0.0040 (0.0541)	−0.1385 (0.1966)
${\hat{\gamma }}_{v}$	*	*	*	−0.1899 (0.0346)	*	*	−0.2110 (0.0408)	−0.4723 (0.0903)
${\hat{\gamma }}_{\vartheta v}$	*	*	*	*	*	−0.1866 (0.0333)	*	−0.2870 (0.0973)
$fra{c}_{{\vartheta }_{1}}$	*	*	30.98	*	*	26.41	28.01	37.45
$fra{c}_{{\vartheta }_{2}}$	*	*	32.17	*	*	*	28.51	41.92

*Non-available.

The correlation parameters *γ*_*υ*_, *γ*_*ϑ*_, and *γ*_*ϑv*_ for Models 4, 5 and 6, respectively, are not significantly different probably because they use similar structures to account for the correlations. Model 6 is an improvement of Models 4 and 5; although the DIC is only slightly lower than the previous models, it is able to capture the fraction of spatially structured variation within the random intercepts. For Models 4 to 8, the correlation parameters can be interpreted as scale factors to estimate the scaled versions of the random intercepts. Specifically, for Model 4, *γ*_*υ*_ = −0.1899 is the scale factor for estimating the random slopes from the corresponding random intercepts. The correlation parameter *γ*_*ϑv*_ for Model 8, on the other hand, can be likened and interpreted as Pearson’s correlation coefficient.

**Figure 1:**
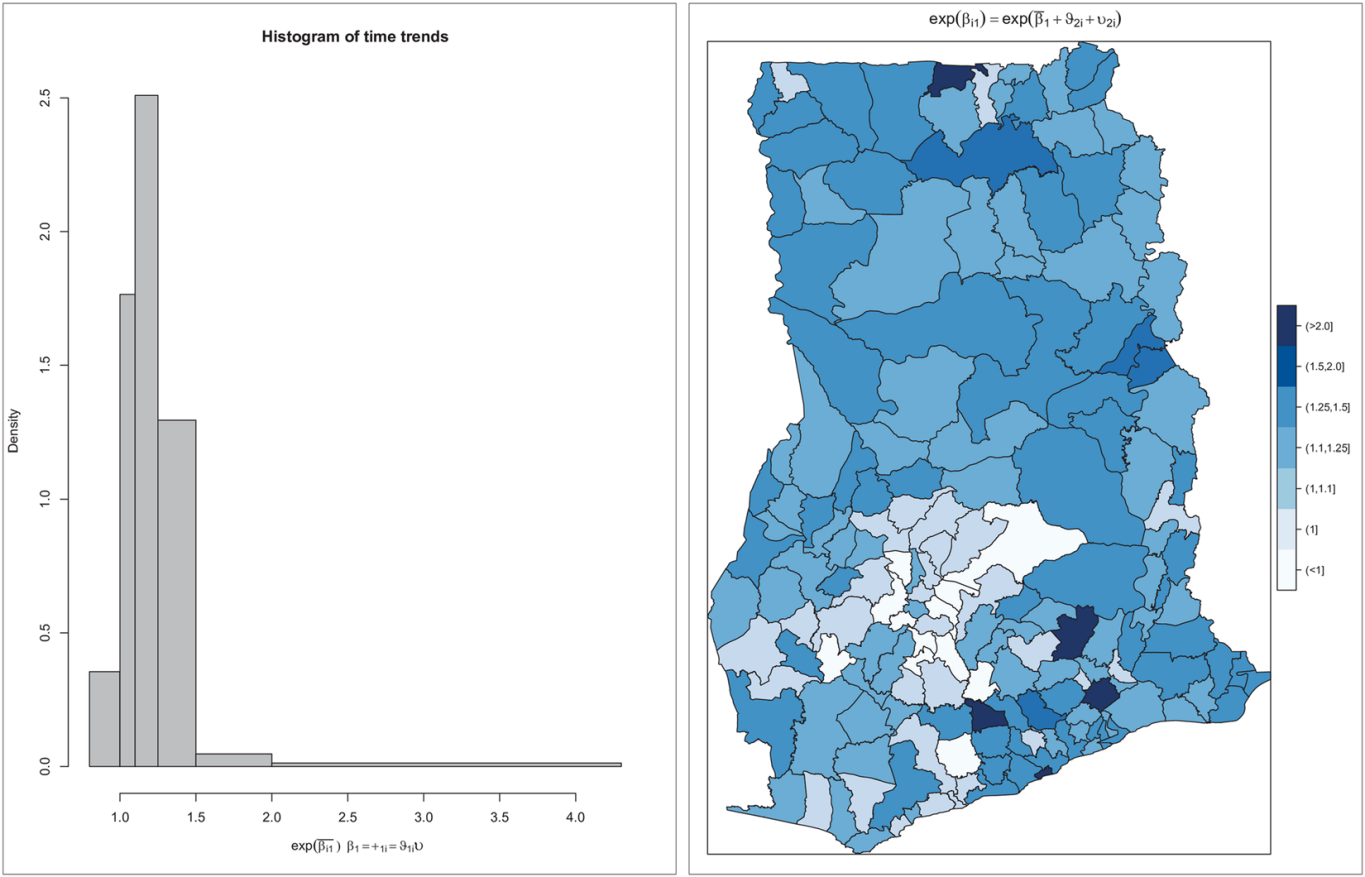
Map of the spatial distribution of the differential time trends (left) and its histogram (right).

**Figure 2:**
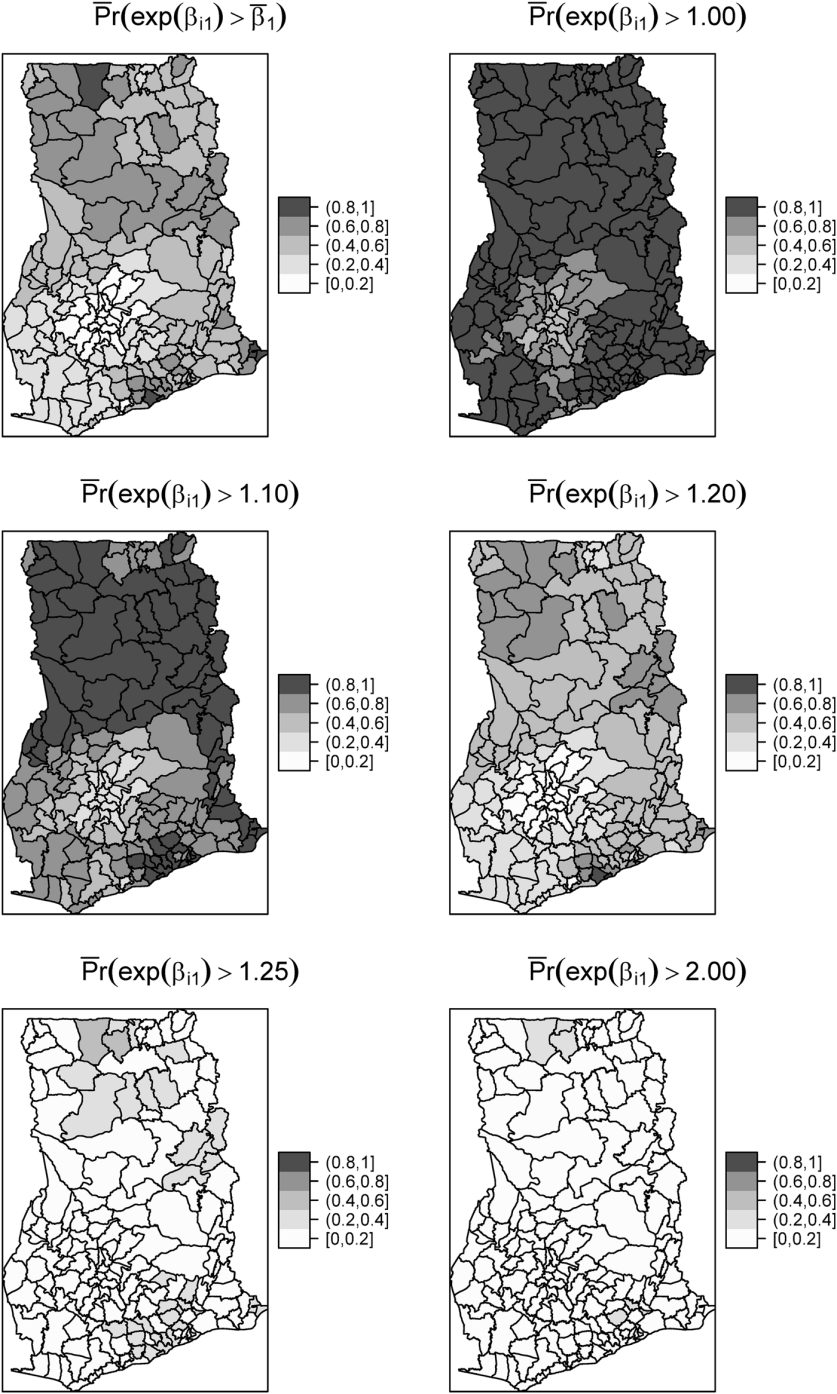
Exceedance probabilities of the differential time trends for different thresholds.

Model 8 shows there is an overall increasing time trend of $\exp ({\bar{\beta }}_{1})=1.23$ for diarrhea in Ghana. This is also interpreted as the rate ratio between two consecutive years and implies diarrhea risk increases 1.23 times every year. The estimate of the correlation parameter *γ*_*ϑv*_ = −0.287 implies that lower intercepts are associated with more positive slopes. We further present maps of the posterior estimates of important quantities. Figure 1 shows the distribution of the differential time trends which is of primary interest. For ease of interpretation, *β*_1*i*_ are exponentiated such that they are interpreted as multiplicative effects. Thus, exp(*β*_1*i*_) > 1 implies a positive time trend, whereas exp(*β*_1*i*_) < 1 implies a negative time trend. We observe increasing trends of diarrhea throughout, except for a few districts within the south-central parts which have a decreasing or no temporal changes. There are also isolated instances (4 districts) with extreme time trends exp(*β*_1*i*_ > 2.0). To assess the statistical significance and the clustering patterns of the time trends, we mapped the spatially smoothed posterior probability that *β*_1*i*_ exceeds the mean time trend ${\bar{\beta }}_{1}$. Same were estimated for the thresholds $\exp ({\beta }_{1}^{Th})=\{1.0,\,1.10,1.20,1.25,2.0\}$ (Fig. 2). Districts with darker (lighter) grey have at least 80% probability of exceeding (falling below) the threshold. While fewer districts have at least a 0.8 chance of their trend to exceed the mean time trend $\exp ({\bar{\beta }}_{1})=1.23$, the majority of districts have 80% chance of their trends to exceed ${\beta }_{1}^{Th}=1.0$, just as that of ${\beta }_{1}^{Th}=1.10$. This suggests an increasing time trend for the majority of the districts. No district has at least 0.8 chance of its trend to exceed $\exp ({\bar{\beta }}_{1})=1.25$ and $\exp ({\bar{\beta }}_{1})=2.0$. This indicates that the few isolated areas identified with extreme time trends exp(*β*_1*i*_ > 2.0) are outliers.

**Figure 3:**
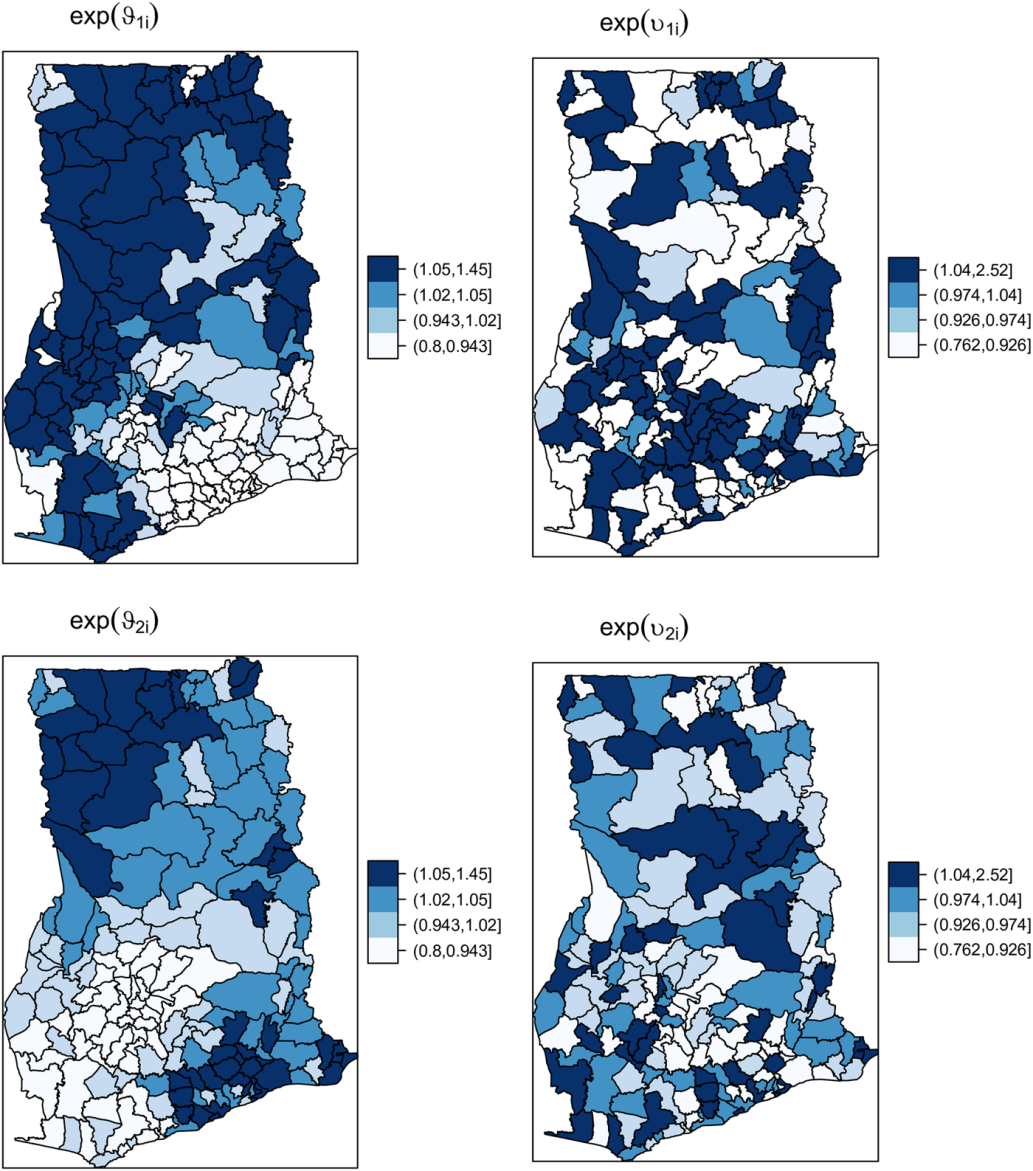
Residual random effects of the structured and unstructured time trends and the risk.

**Figure 4:**
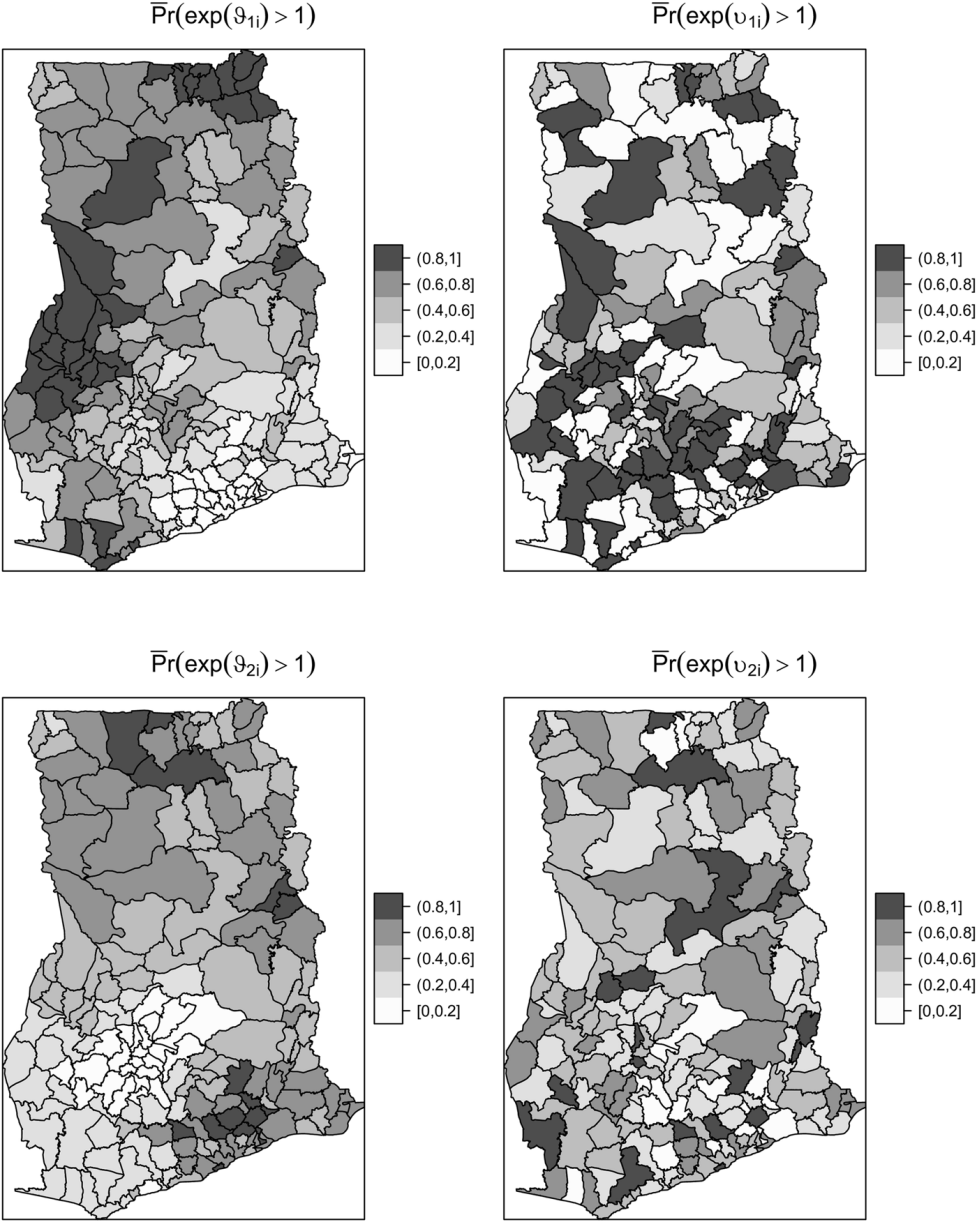
Exceedance probabilities of Residual random effects of the structured and unstructured time trends the risk.

Figures 3 and 4 show the random effects of the intercepts and slopes and their associated exceedance probabilities, respectively. Recounting Fig. 4, maps of exp(*ϑ*_1*i*_) and exp(*υ*_1*i*_) are interpreted as residual spatially structured and unstructured risks after accounting for time trends, respectively. That of exp(*ϑ*_2*i*_) and exp(*υ*_2*i*_), on the other hand, are the residual time trends after accounting for the mean time trend. As expected, we observe no spatial similarity between these maps, except that the unstructured random effects dominated the structured components. Comparing the maps of exp(*ϑ*_2*i*_) and exp(*υ*_2*i*_), we can construe that the spatially unstructured components of the time trends dominated and accounted for 64.8% of the random slope variation (Table 3). Similarly comparing exp(*ϑ*_1*i*_) and exp(*υ*_1*i*_), the spatially unstructured residual spatial effects dominated by 71.9%.

## Discussion

This study presents an extension of a standard approach to analyzing spatial patterns of time trends in spatial epidemiology as has been developed by Bernardinelli *et al*.^17^. Unlike the direct applications of existing methods^17,18^ and their applications, our focus has not only been on estimating the time trends but also evaluating the spatial clustering of the time trends. The epidemiological context of the study dwells on studying the differential time trends of diarrhea occurrences in Ghana. Estimating the time trend for each area would be a critical challenge in frequentist statistics in terms of reliability of estimates due to the number of fewer time stamps. In our hierarchical Bayesian space-time approach, we have taken advantage of the ability to borrow information across both space and time to improve the reliability of our estimates. The inclusion of the correlation term between the random intercepts and slopes has both methodological and epidemiological implications. Models with the correlation terms were observed to be superior, in terms of prediction power, over those without.

Here, we discuss both the issues arising out of the statistical modeling and the epidemiological implications for the analysis of diarrhea surveillance data in Ghana. The preferred model, amongst those without correlation between the intercepts and slopes, is Model 3. This model avoids choosing between heterogeneity and clustering for both the time trends and residual spatial variations. The significance of the correlation parameters in Models 4 to 7 suggest the importance to include correlation between the intercepts and slopes, the avoidance of which would cause estimates of the district-specific trend to be pulled towards the mean trend^17^. Derivation and comparing variant extensions to incorporate this correlation proofed worthwhile as the strengths and weakness of each approach were unveiled. Inducing correlation by expressing the random components of the slopes as a linear function of the random intercepts is straightforward. Yet, the model with BiCAR correlation (Model 8) showed higher performance and preferred over the others in our case. This model has the advantage to estimate the correlation between the unstructured intercepts and slopes, between the structured intercepts and slopes, and the overall correlation. Important differences in our models are the variation in time trends and the way correlation between intercepts and slopes are accommodated. We conclude that the inherent structure of the data should guide the choice of correlation method by comparing competing models. Centering the covariate, in this case, the time, should reduce the correlation between the intercepts and slopes, but not for the case of strong inherent correlation. For cancer related and non-infectious diseases this might work. In our case, we explicitly accounted for the inherent correlation. We observed the correlation to be significant even though the time variable was centered. Robust parameter estimation is not the only advantage to account for correlation; the kind of correlation between the random intercepts and slopes (either negative or positive) presents an important etiological clue regarding the response of the population to environmental or climatic changes. For our study, Models 4 to 8 indicated a negative association between the intercepts and slopes, implying that the district-specific risks are converging to the same levels. The etiological clue might be that environmental risk factors which were frequently different in the past are currently frequently similar. Population growth, unplanned urbanization, and rural-urban migration which have exceeded the availability of safe drinking water and sanitation could be the causal factors.

From an epidemiological point of view, the current study helps to form a clearer picture of the space-time trends of diarrhea in Ghana, prompting for an effective control strategy. The average time trend, $\,exp({\bar{\beta }}_{1})\approx 1.23$, reflecting a yearly incremental rate of 23% is striking as it far beats the yearly population growth rate of nearly 2.3%. This alone is enough indication to prompt health officials and policymakers about the severity of diarrhea menace. Returning to the observed spatial patterns of the time trends which is the primary objective of this study (Fig. 3), there is wide spatial variation, of which the unstructured components dominate over the structured ones. This is where avoiding choosing between heterogeneity and clustering is relevant. It suggests the importance of household level risk factors (captured by unstructured heterogeneity) in controlling the temporal effects of diarrhea. The spatially varying growth rates could indicate varying responses to time-varying climatic risk factors such as temperature, humidity, and rainfall. These are known to influence the risk of diarrhea infections^10,38^. Additional plausible interpretation, especially for districts with high time trends, could be improvements in the disease surveillance systems or reporting strategies. Additionally, the random effects of the time trends, especially the structured components, may also be concomitant with district-level population growth changes, but further studies are required to sustain this as fact. For the unstructured component, deterioration of household-level water and sanitation amenities are plausible factors and likewise requires further studies. That said, the probability maps of the structured and unstructured time trends could easily support policymakers about districts of needed concern for either household level targeting or regional (beyond district-level) targeting. Either way, improvements in household level amenities will have ripple effects on district or regional-level clustering. In a nutshell, these findings deserve further reflection by health officials and policymakers in line with which prompt actions might be required.

Most small area health studies focus on small area contrasts of the risk for a single temporal window or temporal contrasts for the whole study area. While we developed our models with a focus on diarrhea in Ghana, an extension to other diseases and/or other developing countries is conceptually straightforward. The novelty of our study is partly based on the variant approaches developed to induce correlation between the intercepts and slopes. Here, for our data with few times stamps, we have used Bayesian random effects model to evaluate the small area contrasts of the linear temporal trends. It can be argued that our approach is similar to the Type IV interaction since the linear trends (structured) with local slopes are specified for each district which are then further smoothed (structured) over space, except that we additionally focused on the spatial clustering of the time trends. Also, accounting for correlation between the intercepts and slopes is therefore simple since each spatial units has a single slope unlike the case of the Type IV where the number of slopes per spatial unit is equivalent to the length of the random walk. However, for data with many time stamps, our approach has the disadvantage to over smooth the temporal trends due to the imposition of linear time trends. Extension of the parametric linear trend model to a nonparametric time trend model like the random walk approach, however, is possible when the focus is to detect where and when the time trends cluster.

In a similar objective to our study, Waller *et al*.^39^ explored the spatio-temporal patterns in the county-level incidence of Lyme disease in the northeastern United States. Another distinction here is that we have developed and compared different methods to accommodate the inherent correlation between the random components of the intercepts and slopes. It may be possible to extend the time trend component to a random walk prior instead of imposing linearity if data for a substantial number of temporal points are available. Within our study area, a typical infectious disease extension is to compare with the spatially varying temporal trends of intestinal parasites morbidities which also has similar risk factors. In the future, we intend to extend our study to a bivariate model, where the spatially varying time trends of two diseases could be determined jointly.

Now we turn to the implications of our study for public health policy and interventions. We indeed reiterate that, in health policy research, the models could be implemented to identify and map areas with extremely increasing time trends with a view of determining critical areas needing interventions. In fact, it is noteworthy to state that the patterns of infectious diseases are dynamic rather than static. Hence, since our study is retrospective, public health intervention policies should rather be based on results of new data at the time of their developments.

Our study also has some limitations. First, our study has the implicit assumption of equal within-district time trends without consideration for household or relatively smaller area-level trends. Although we attempted to capture this effect through the unstructured random components of the time trends, it could instead be spurious. That said, the significance of the study to public health far outweighs these limitations. In the future, we intend to focus on spatial disaggregating of the time trends to account for within area variation.

## Conclusions

In this study, we have demonstrated how space-time random effect modeling is useful to detect district-specific time trends of diarrhea. Models which account for correlation between varying intercepts and slopes are competitive than those without, with a BiCAR method being the most competitive. The inclusion of correlation between the intercepts and slopes provided additional epidemiological information, illuminating the response of the disease dynamics to environment changes in past and present. We found increasing trends of diarrhea risk amongst many districts, but many with trends lesser than the overall mean trend. The spatially varying trend maps are useful for guiding interventions and resource allocations geared towards curbing this menace. Extensions of our models to other or multiple infectious diseases are straightforward, and we seek to venture into this in the future. However, flexible usability of our approach by public health professionals in Ghana will require further engagements and capacity training which we aspire to fulfill in the future.

## Data Availability

The datasets used and/or analyzed during the current study are available from the CHIM of the Ghana Health Services upon reasonable request
